# Dietary *Chlorella vulgaris* Ameliorates Altered Immunomodulatory Functions in Cyclophosphamide-Induced Immunosuppressive Mice

**DOI:** 10.3390/nu9070708

**Published:** 2017-07-06

**Authors:** Dai Cheng, Zhaodong Wan, Xinyu Zhang, Jian Li, He Li, Chunling Wang

**Affiliations:** 1 Beijing Advanced Innovation Center for Food Nutrition and Human Health, Beijing Technology & Business University (BTBU), Beijing 100048, China; dcheng@tust.edu.cn (D.C.); lijian@th.btbu.edu.cn (J.L.); lihe@btbu.edu.cn (H.L.); 2 Key Laboratory of Food Safety and Sanitation, Ministry of Education, College of Food Engineering and Biotechnology, Tianjin University of Science and Technology, Tianjin 300457, China; wzdtust2014@126.com (Z.W.); ZhangXY_0219@163.com (X.Z.)

**Keywords:** *Chlorella vulgaris*, cyclophosphamide, immunosuppressive mice, diet

## Abstract

Based on the well-known toxicity of cyclophosphamide (CYP) on the immune system, this research investigated the modulating effects of the long-term dietary *Chlorella vulgaris* (CV) supplementation on the immunosuppression induced by CYP in mice, in order to provide a novel dietary design to mitigate the side effects of CYP therapy. Control, CYP-treated, CYP + CV (6%), CYP + CV (12%) and CYP + CV (24%) were used for 6 weeks, CV supplement in diet recovered the significantly reduced immunological function in CYP treated mice. As CV may have a modulating function through the inducible expression of cytokines, we assayed the expressions of interleukin-2 (IL-2), interleukin-12 (IL-12), tumor necrosis factor-α (TNF-α) and interferon-γ (IFN-γ). Our results suggested that CYP significantly reduced the lymphocytes proliferation and phagocytic activities of macrophages, and stimulated the production of IL-2, IL-12, TNF-α and IFN-γ and that this impairment has been successfully adjusted by CV supplementation. Treatment with the algae also enhanced the natural killer (NK) cells cytotoxicity, and ameliorate histological changes of the spleen in CYP-treated mice. Therefore, as we found in this study, a diet supplemented with whole CV has beneficial effects on CVP-induced immunosuppression, through its immunomodulatory potential.

## 1. Introduction

Chemotherapeutic drugs have been widely used in the treatment of various malignancies and autoimmune diseases [1], but their therapeutic effectiveness is accompanied with some severely adverse side effects [2]. Cyclophosphamide (CYP) is an alkylating cytotoxic drug and has been applied in the therapy of several cancers and autoimmune disorders, such as rectum cancer, liver cancer, lymphoma, breast cancer and rheumatoid arthritis [3]. However, patients undergoing CYP therapy must face some severe adverse effects including immunosuppression and oxidative stress injuries [4]. CYP, particularly when used in high-dosage and for long periods, could lead to a decline in body weight, splenocyte proliferation, organ index, macrophage phagocytosis and natural killer (NK) cell activity in reported experiments [5,6]. Thus, it is important for patients to avoid the damage of immunosuppression during CYP treatment.

A growing number of studies have shown that nutrition supplement and dietary change are deemed critical to regulating immunity response [7,8,9]. *Chlorella vulgaris* (*Chlorophytes, Chlorophyceae*), a unicellular green alga, is a potential health food source with a proportional content of many macro- and micronutrients including proteins, essential amino acids, carbohydrates, dietary fibers, fatty acids, nucleic acids, vitamins, growth factors, minerals, and chlorophyll [10]. *Chlorella vulgaris* (CV) is widely utilized in Japan, the USA, Europe, and other countries [11], especially in East Asia, where it is consumed with rice, tea and pancakes [12]. Previous researches proved that CV and its extracts ameliorate physiological health conditions, e.g., by improving the immune function [13,14], regulating lipid metabolism or tumors [15], motivating dioxin excretion [16], and normalizing physiological function [17]. It has been shown that oral treatment of CV extract increases the level of of interleukin-2 (IL-2) mRNA in the spleens and macrophages in mice [18]. Moreover, CV enhanced the levels of interferon-γ (IFN-γ) mRNA in the spleens of common mice and murine syndrome of acquired immunodeficiency mice [19].

Considering literature data, as an integrated food, the CV would be an abundant element of a bioactive diet. Therefore, the present study investigated the protective effect of the 6-week supplementation with CV on the immunosuppression induced by CYP in vivo in an experimental model. In addition, the activities of relevant enzymes (lactate dehydrogenase and acid phosphatase), the levels of cytokines (IL-2, IL-12, tumor necrosis factor-α (TNF-α) and IFN-γ), pathomorphology of splenocyte, macrophage and NK cell activities were measured to evaluate the conceivable immune enhancing functions.

## 2. Methods and Materials

### 2.1. Materials

The *Chlorella vulgaris* (CV) used in this study were prepared to lyophilize the heat-treated CV, supplied by the Tianjin Key Laboratory of Marine Resources and Chemistry, College of Marine Science and Engineering, Tianjin University of Science and Technology, Tianjin, China. The primary nutritional composition content of the CV powder is shown in Table 1.

### 2.2. Animal Model and Diets

Male Kunming mice that were eight weeks old, 30 ± 5 g, and clean grade, were used. The experiments were carried out according to the Animal Management Rules of the Ministry of Health of the People’s Republic of China (documentation No. 55 (2001), Ministry of Health of P.R. China), with utilization permission from the Animal Department of the Academy of Military Medical Sciences, SCXK (Jun) 2007-004. All mice were kept in a temperature-controlled environment (25 ± 2 °C) at 60 ± 5% relative humidity, with a 12 h (dark)-12 h (light) cycle and allowed free access to water and food for 7 days before the experiment, and were randomly distributed among 5 groups for various treatments (Table 2). After the last feed, mice fasted for 24 h. Five mice in each group were weighed, then sacrificed by cervical decapitation. The spleen and thymus tissues were excised immediately, washed in ice-cold isotonic saline, blotted with filter paper, and weighed.

Spleen samples were sliced and fixed in a 10% formalin solution. The specimens were then embedded in paraffin and sliced into 5 μm thick sections, which were then stained with hematoxylin–eosin. The sections were examined by an experienced observer who was blind to the treatment under the light microscope after which photomicrographs were taken.

### 2.3. Assay of Splenocyte Proliferation

A fraction of the spleen tissue was used for the preparation of splenocytes. The cells were away from erythrocyte by treatment with lysis buffer (Sorlabio Co., Beijing, China). To eliminate adherent cells, the splenocytes were cultivated in 6-well plate (Corning Inc., Corning, NY, USA) for 3 h.

The splenocyte proliferation was evaluated by performing the Methylthiazolyldiphenyl-tetrazolium bromide (MTT) method. A volume of 100 μL of splenocytes (1 × 10^5^/mL) was sowed in a 96-well plate (Corning Inc., Corning, NY, USA), then the concanavalinA (ConA) (Solarbio Co., Beijing, China) and lipopolysaccharide (LPS) (Solarbio Co., Beijing, China) (5 μg·mL^−1^) were added to a final volume of 200 μL. The incubation was in an incubator with 5% CO_2_ for 48 h at 37 °C. After that, 20 μL of MTT (1 mg/mL) was added to each well and incubated for 4 h. Each plate was centrifuged (1000 r·min^−1^ for 5 min) after which the supernatant was abandoned, and dimethylsulfoxide (DMSO) (150 μL) was added to each well for 1 h. The absorbance at 570 nm was detected using a microplate ELISA reader (Thermo Fisher, Waltham, MA, USA).

### 2.4. Assay of NK Cell Cytotoxicity

YAC-1 (Mouse lymphoma cell lines) cells purchased from the Shanghai Institute of Cell Biology (Chinese Academy of Sciences, Shanghai, China) served as the objective cells for NK cell cytotoxicity evaluation. The splenocytes were co-cultured with YAC-1 cells at a ratio of 25:1 in 96-well plates. The incubation was in an incubator with 5% CO_2_ for 5 h at 37 °C. 50 μL MTT (2 mg/mL) was added to each well and incubated for 4 h after which 150 μL DMSO was added to each well for 1 h. The absorbance at 570 nm was detected using a microplate ELISA reader (Thermo Fisher, Waltham, MA, USA). NK cell cytotoxicity was computed as the equation: NK cytotoxicity = (A1 − (A2 − A3))/A1, where A1, A2, and A3 are absorbance of target cells control, test samples, and effector cells control, respectively [20].

### 2.5. Biochemical Assessment

One portion of the spleen was homogenized in ice-cold saline (1:10, *w*/*v*); the homogenate was centrifuged (10,000× *g* at 4 °C for 30 min) and the supernatant was used for the estimation of biochemical parameters. The activity of lactate dehydrogenase (LDH) and acid phosphatase (ACP) were estimated using reagent kits (Nan Jing Jian Cheng Bio Institute, Nanjing, China).

### 2.6. Isolation of Peritoneal Macrophages

After the last feed, five mice were utilized in each group for macrophage donation. Peritoneal exudate cells (hereafter termed macrophages) were collected from the peritoneal cavities of mice, which had been injected intraperitoneally with 3 mL of thioglycollate for three days before peritoneal lavage with 10 mL of Hank’s balanced salt solution (Sigma, St. Louis, MO, USA).

The survivability of separated cells was measured by trypan blue exclusion, and the ratio of macrophages was measured by the observation of cytoplasm stained with acridine orange by a fluorescence microscope. Cell preparations were >95.5% viable and contained >90% macrophages [7].

### 2.7. Macrophages Viability Assay

The MTT assay was performed to evaluate the survivability of macrophages. Macrophages (1 × 10^5^ cells per mL, 200 μL) were sowed in 96-well microculture plates. The incubation was in an incubator with 5% CO_2_ for 48 h at 37 °C. After that, 20 μL of MTT (1 mg/mL) was added to each well and incubated for 4 h. Each plate was centrifuged (1000 r·min^−1^ for 5 min) and the supernatant was abandoned, DMSO (150 μL) was added to each well for 1 h. The absorbance at 570 nm was detected using a microplate ELISA reader (Thermo Fisher, Waltham, MA, USA).

### 2.8. Neutral Red Uptake by Macrophages

A volume of 100 μL of macrophages (1 × 10^5^ cells per mL) were sowed in 96-well plates, and 100 μL/well of neutral red (0.075%) was added. Then the macrophages were incubated at 4 °C for 4 h and washed with ice-cold PBS three times. After that, cell lysing solution (100 μL) was added, and the cells were incubated at 4 °C for 2 h. The absorbance was measured at 540 nm in a microplate reader [21].

### 2.9. Reverse Transcript-PCR Analysis of Interleukin, TNF-α and IFN-γ mRNA

The splenocyte cells and macrophages were prepared as above respectively. The total RNA from 1 × 10^6^ were extracted with 1 mL Trizol reagent (Transgen Biotech. Co., Beijing, China), and the concentration of the total RNA was reversely transcribed using RT-PCR kits (Transgen Biotech. Co., Beijing, China). The reaction was performed at 42 °C for 30 min and 85 °C for 5 min to prepare cDNA, which was then stored at −20 °C until use.

The PCR was performed on the revers-transcribed cDNA product to determine the expression levels of IL-2, IL-12, IFN-γ, TNF-α and β-actin (as an internal control). The reactions were performed using the RT-PCR kits (Transgen Biotech., Co., Beijing, China). After an initial pre-denaturation for 4 min at 94 °C, an amplification sequence protocol of denaturation for 30 s at 94 °C, annealing for 30 s at 60 °C, and extension for 45 s at 72 °C for 35 cycles was used, followed by a final extension step for 10 min at 72 °C. The primers used in the present study are listed in Table 3.

### 2.10. Statistical Analysis

The results are the mean ± standard deviation (SD) of triplicates, analyzed using SPSS 17.0 (IBM, Armonk, NY, USA). The statistical significance of data comparisons was assayed using one-way analysis of variance (ANOVA) followed by Dunnett Contrast Fits. Values for *p* < 0.05 were considered statistically significant.

## 3. Results

### 3.1. Effects of CV on Thymus and Spleen Indices in CYP-Induced Mice

**Figure 1:**
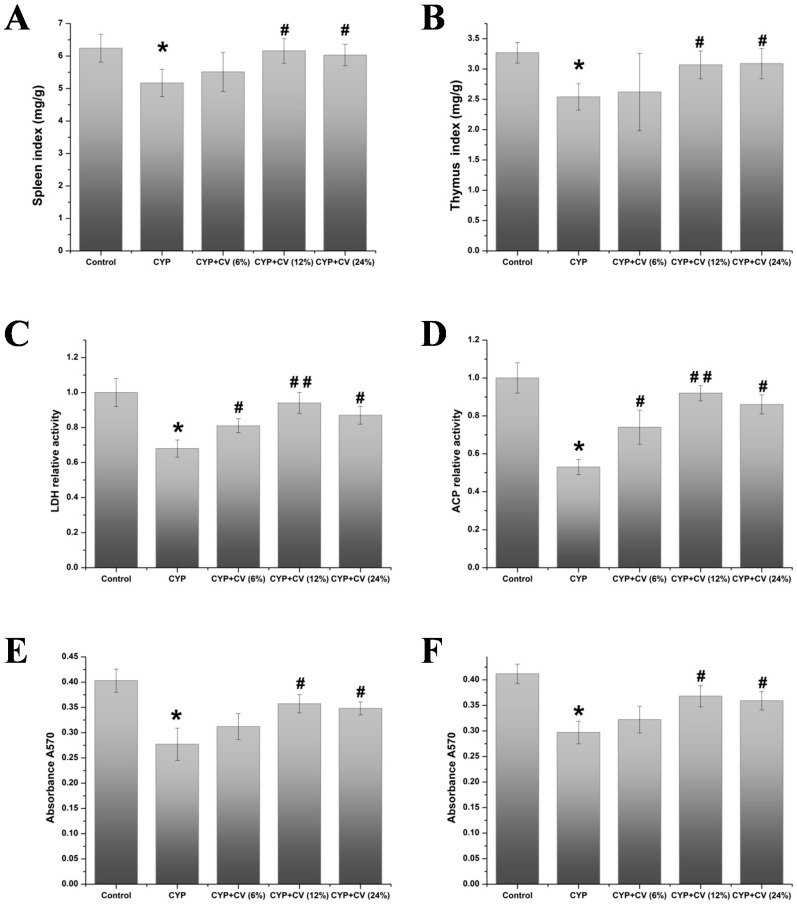
Effect of *Chlorella vulgaris* (CV) on the spleen index (**A**), thymus index (**B**), the relative activity of lactate dehydrogenase (LDH) (**C**), acid phosphatase (ACP) (**D**), splenocyte proliferation added with concanavalinA (ConA) (**E**) and lipopolysaccharide (LPS) (**F**) in the cyclophosphamide (CYP)-treated mice. Note: Data were expressed as mean ± standard deviation (SD), *: only the CYP treatment was compared against the control treatment (*p* < 0.05); #: only the CYP + CV treatment was compared against the CYP treatment (*p* < 0.05), ##: only the CYP + CV treatment was compared against the CYP treatment (*p* < 0.01).

The amount of daily food intake was roughly equal to the control, CYP, CYP + CV (6%, 12%, 24%) diet-fed mice: 5 ± 0.5 g in all groups. The growth rate, calculated by measuring the body weight weekly, did not show a significant difference between the five groups (data not shown). Relative organ mass is an important index for evaluating the toxicity of organs. Effects of CYP and CV on spleen and thymus indices in the experimental mice have been shown in Figure 1A,B. Compared with the control mice, the spleen and thymus indices were significantly decreased in the mice treated with CYP; however, the two organ indices were significantly increased in the mice whose diet had been supplemented with CV, except the CV (6%) treated group. The experiment suggests that CV reduces the immunosuppression in CYP-treated mice.

### 3.2. Effects of CV on the Relative Activity of LDH and ACP in Spleen in Mice

As shown in Figure 1C,D, the decline of LDH and ACP was observed in CYP-treated mice. On the other hand, supplement of CV in CYP-injected mice showed significant restoration in the activity of ACP and LDH in spleen tissue, and this showed that CV could modulate the immunological function of the spleen in an immunosuppressed state induced by CYP.

### 3.3. Effects of CV on Splenocyte Prolife Ration and Natural Killer Cell Cytotoxicity in Mice

**Figure 2:**
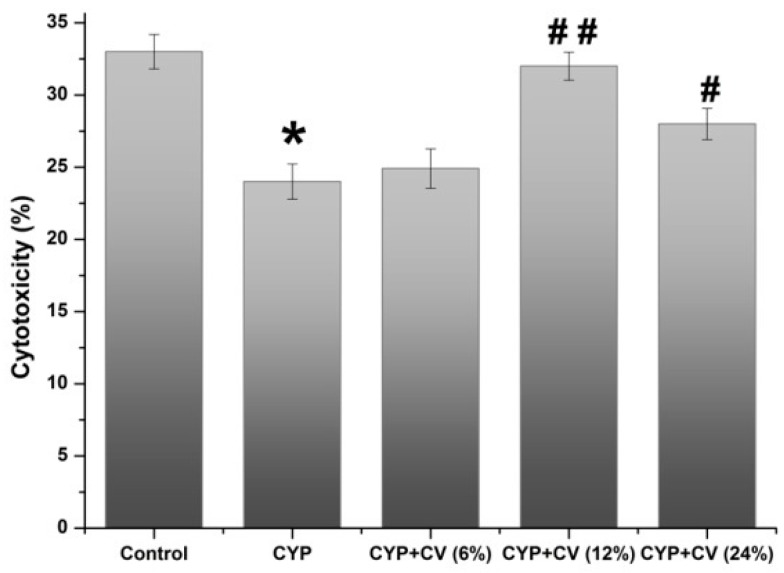
Effect of CV on the natural killer (NK) cell cytotoxicity in the CYP-treated mice. Note: Data were expressed as mean ± SD, *: only the CYP treatment was compared against the control treatment (*p* < 0.05); #: only the CYP + CV treatment was compared against the CYP treatment (*p* < 0.05), ##: only the CYP + CV treatment was compared against the CYP treatment (*p* < 0.01).

As shown in Figure 1E,F, the proliferation of splenocytes of the CYP group was decreased obviously compared with the control group. Combing with LPS or ConA, CV increased the proliferation of splenocytes. CV at 12% and 24% additive amount showed obvious motivation in the proliferation of splenocytes with ConA or LPS. The effects of CV on natural killer cell cytotoxic activities in CYP-treated mice were measured (Figure 2). Compared with the CYP group, the natural killer cells cytotoxicity of CV groups were increased strikingly, except the CV (6%) treated group.

### 3.4. Histopathological Analysis of Spleen in CYP-Treated Mice

**Figure 3:**
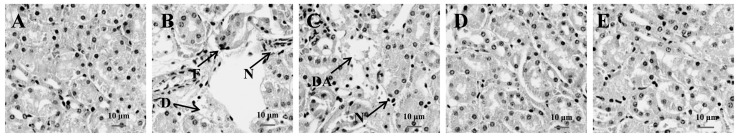
Photomicrograph of spleen from each experimental group (magnification = 100×). (**A**), control group, normal diet, showing the absence of damage of spleen tissue; Compared with the control, the CYP group (**B**) showed evident necrosis region (N), cellular fibrosis (F) and disordered arrangement of cells (DA). In the CV (6%) group (**C**), the necrosis region became minor and the intercellular space was lessened as compared with the CYP group. The CV (12%) group (**D**) and CV (24%) group (**E**) had unbroken cell, and fibrosis cells became smaller.

As Figure 3 showed, control group (A) showed a normal appearance of spleen tissue. Compared with the control, the CYP group (B) showed evident necrosis region, cellular fibrosis and disordered arrangement of cells. In the CV (6%) group (C), the necrosis region became minor and the intercellular space was lessened as compared with the CYP group. The CV (12%) group (D) and CV (24%) group (E) had an unbroken cell, and fibrosis cells became smaller. This indicated that the protective role of CV on spleen in immunosuppressed state was induced by CYP.

### 3.5. Effects of CV on Proliferation and Phagocytosis Activity of Macrophages in Mice

**Figure 4:**
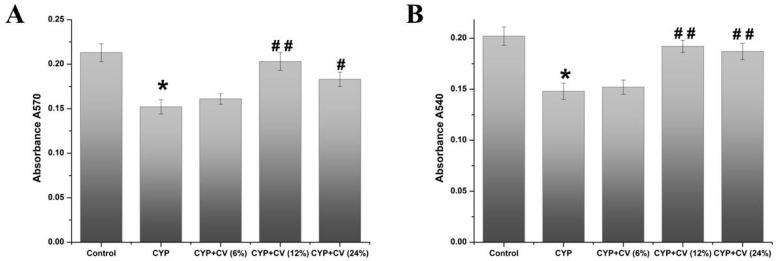
Effects of CV on proliferation (**A**) and phagocytosis activity (**B**) of peritoneal macrophages in CYP-treated mice. Note: Data were expressed as mean ± SD, *: only the CYP treatment was compared against the control treatment (*p* < 0.05); #: only the CYP + CV treatment was compared against the CYP treatment (*p* < 0.05), ##: only the CYP + CV treatment was compared against the CYP treatment (*p* < 0.01).

An obvious enhancement of proliferation has been observed in the groups treated with CYP + CV 12% or 24%, respectively, when compared with the CYP group. CV seemed to enhance the proliferation activity of macrophages. In the present study, the effect of CV on macrophages phagocytosis of CYP-treated mice was evaluated by the neutral red assay. As shown in Figure 4B, CV had a higher absorbance than the CYP group at a concentration range of 12–24%, and absorbance was enhanced in a dose-dependent manner. These results showed that treatment of CV might trigger an immune response.

### 3.6. Effect of CV on the Expression of Cytokines (IL-2, IL-12, TNF-α, IFN-γ) of Splenocytes in Mice

**Figure 5:**
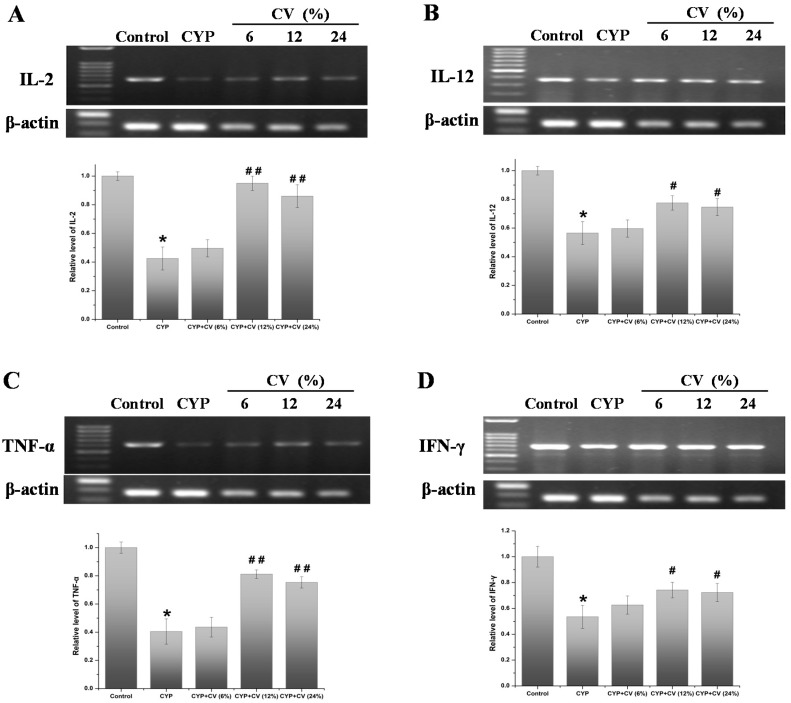
Effects of CV on the mRNA expression levels of IL-2 (**A**), IL-12 (**B**), TNF-α (**C**) and IFN-γ (**D**) in the spleen of CYP-treated mice. The β-actin was used as the control. Note: Data were expressed as mean ± SD, *: only the CYP treatment was compared against the control treatment (*p* < 0.05); #: only the CYP + CV treatment was compared against the CYP treatment (*p* < 0.05), ##: only the CYP + CV treatment was compared against the CYP treatment (*p* < 0.01).

**Figure 6:**
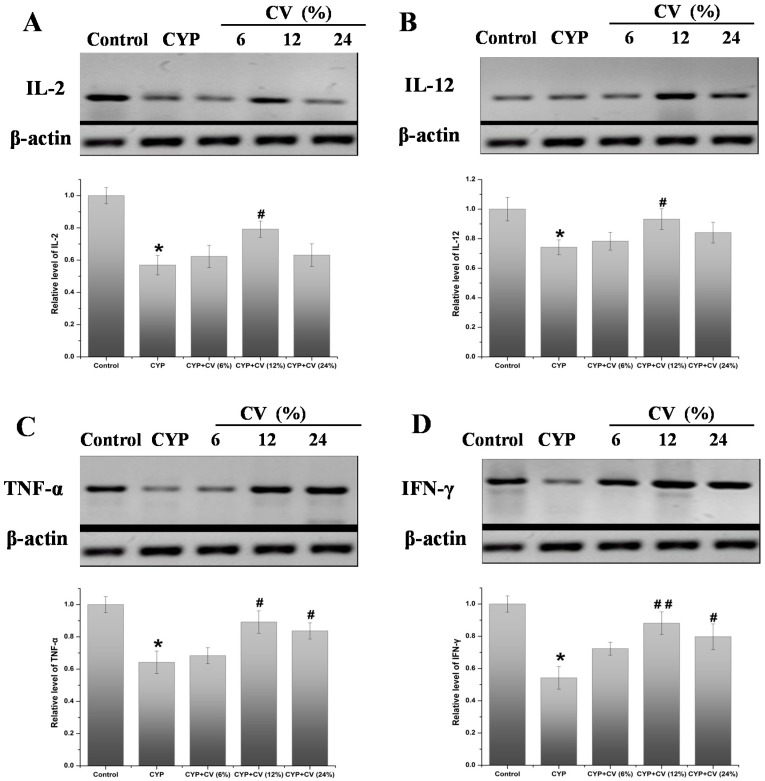
Effects of CV on the level of IL-2 (**A**), IL-12 (**B**), TNF-α (**C**) and IFN-γ (**D**) proteins in the spleen of CYP-treated mice. The β-actin was used as the control. Note: Data were expressed as mean ± SD, *: only the CYP treatment was compared against the control treatment (*p* < 0.05); #: only the CYP + CV treatment was compared against the CYP treatment (*p* < 0.05), ##: only the CYP + CV treatment was compared against the CYP treatment (*p* < 0.01).

As shown in Figure 5, CV-enriched diets show the effects on the expression of cytokines in splenocytes of experimental mice. The mRNA levels of IL-2, IL-12, TNF-α, IFN-γ in CYP group were decreased as compared to the control group. The CV groups showed significantly increased mRNA levels of these cytokines. Compared to the CYP treated group, these cytokines mRNA levels in splenocytes of CV 12% treated group were enhanced significantly (*p* < 0.01), but the groups treated with CV 6% showed lower expression levels, compared with the normal group and similar levels of the CYP group. These cytokines proteins levels in spleen were analyzed by the Western blot method. As shown in Figure 6, the CV 12% treated group shows the best exaltation level, while the effect of CV 6% or 24% is not noticeable. These results showed that CV treatment could change the down-regulation expressions of mRNA and protein induced by CYP, but the dose-dependence was not linear.

## 4. Discussion and Conclusions

CYP is an effective chemotherapeutic drug widely used as an immunosuppressant in the treatment of various human cancers and rheumatoid arthritis as well [22]; however, its serious and evident toxicity on target organs and common cells is worrisome [23]. Thus, there is urgency for an efficacious chemopreventive agent or food supplement which can decrease the toxicity of CP therapy. The purpose of the present study was to find the possibly immunomodulatory effect of CV against immunosuppression induced by CYP. It was shown that supplementation with CV improves the function of immune cells in mice that had their immune function compromised with CYP, which might be through avoiding the changes in spleen injury and activating macrophages. These findings show a novel biological activity for the popular functional food, CV in the protection of immunosuppressive complications induced by CYP. The CV (12%) supplement significantly alleviated CYP-induced immunosuppression by promoting the host immune response when compared to the CYP group. There are some studies using *Lactobacillus plantarum* or *Lactobacillus casei* as an immunomodulator in CYP-treated mice [24,25], these probiotics had the potential ability to enhance intestine mucosa immunity. Also, with other dietary active ingredients such as polysaccharide [3] and Omega-3 fatty acids [26], all these dietary ingredients administration alleviate the immunosuppression and inflammation caused by CYP treatment to some extent. When compared with these nutrients, a few researches focused on the evaluation of the immunomodulatory effect of the whole food supplement on CYP induced toxicity.

Immunocompromised mice induced by CYP treatment were used as an animal model for these observations [27]. As expected, the administration of 40 mg/kg of CYP for four days to normal immunocompetent mice could engender a state of immunosuppression. CYP administration induced an apparent reduction of organ indices, splenocyte proliferation, phagocytic activity of macrophages and decreased splenic NK cells activities. Moreover, PCR studies demonstrated that CYP treatment could cause a significant reduction of IL-2, IL-12, TNF-α, IFN-γ levels. These results of CYP treatment are in accordance with previous reports [20,28].

The immune system is a host defense system that includes many biological structures and processes within an organism that protects against disease. Disorders of the immune system can lead to inflammatory diseases, autoimmune diseases and even cancer [29]. The spleen plays an important role in terms of maturation and lymphocyte homing and regulates the functions of the immune system [30]. Lymphocyte proliferation is a crucial process in the activation of an adaptive immune system [31]. More recently, T and B lymphocytes proliferation response to mitogens has been widely employed for the analysis of lymphocytes responsiveness. It is generally known that T and B lymphocytes proliferation can be induced by ConA. It has been observed that at lower concentrations (0.1 to 100 ng/mL), LPS could cause a decrease in pinocytosis in both macrophages and monocytes, whereas at higher LPS concentrations, it could enhance pinocytosis in macrophages [32]. As an important step to realizing the mechanism of the immunoregulatory effect of CV, we assayed its functions on lymphocyte proliferation in immunosuppressed mice. The proliferation assay indicated that LPS- and ConA-induced splenocyte proliferation was significantly increased by CV diet, as compared to the CYP group. These results showed that CV is conducive to the activation of lymphocyte proliferation, and the enhancement of the adaptive immune system.

Macrophages play a critical role in innate immune response, and are involved in protecting the host by phagocytosis, serving as antigen-presenting cells to lymphocytes, and releasing numerous cytokines [33]. The immune function could be reflected by the phagocytosis of macrophages to some extent [34]. The phagocytosis of the macrophages in CV-enriched (12% and 24%) diet fed mice was enhanced when compared to CYP-treated mice. The results suggested that CV treatment contributed to a significant improvement of the phagocytic function of macrophages of the mice, indicating that CV supplementation can effectively antagonize the immunosuppression caused by CYP.

Cytokines play a fundamental role in the regulation of the immune response [35], specifically in host responses to infection, inflammation, immunity and cancer [36]. Interleukin plays a major role in promoting proliferation and differentiation of B lymphocytes and in regulating NK cell cytotoxic activities [37]. Studies from our laboratory and others have demonstrated that CV may have a direct myelostimulating effect through the induction of endogenous cytokine production [38,39]. In our experiment, supplementation with CV increased the mRNA expression of IL-2 and IL-12 in spleen and macrophages as compared with the CYP-treated group. Besides, as compared to CYP-treated mice, the mRNA expression of TNF-α and IFN-γ in the spleen of CV-enriched diet fed mice was increased significantly. These results showed that CV supplement could improve the immune function by promoting the expression of correlated cytokines.

Indeed, various in vivo studies on CV have revealed that they exhibit antioxidative [40], hepatoprotective [41], antibacterial [16], and antitumor activity [42]. An increasing number of studies have demonstrated that CV and its extracts enhance the immune function in vitro and in vivo [43,44,45]. Furthermore, some researchers have focused on the immunomodulation of the protein hydrolysate from CV, and found that CV protein hydrolysate stimulated both humoral and cell mediated immune functions positively, such as T-dependent antibody response and the reconstitution of delayed-type hypersensitivity response [46,47]. Hence, in the present study, CV-mediated immunoprotection should not be attributed to the contribution of a particular nutrient. We hypothesize that CV protects the immune system through the combined action of its various nutrients and improves the host immune response. Furthermore, we anticipate that our assay will provide a new focus on the design of strategies to prevent autoimmune diseases, such as Crohn’s disease [48].

In summary, our study suggests that a CV diet exhibits chemoprotective effects against CYP-induced immunosuppression in mice. A possible mechanism for the protection may be attributed to its immunomodulatory potential. CV supplementation can improve the lymphocytes proliferation and phagocytic activities of macrophages, stimulate the expressions of cytokines, enhance the NK cells cytotoxicity, and ameliorate histological changes of the spleen. Therefore, as we found in this study, ingestion of a certain amount of CV in daily diet might be an effective way to prevent immune disorders and might potentially be beneficial for the dietary recommendations for patients undergoing immunosuppressive therapies.

## Figures and Tables

**Table 1:** Nutrient content of *Chlorella vulgaris* powder.

Nutrient Content	CV
Total crude protein (%)	54.6
Total crude starch (%)	19.4
Total crude fat (%)	9.4
Pigmenta (%)	2.9
Ash (%)	5.9
Moisture (%)	7.8

CV: *Chlorella vulgaris*.

**Table 2:** Mice of each group with various treatments.

Group	Treatment	Duration
Control	normal salt diet	42 straight days
CYP	CYP 40 mg·kg^−1^ (0.2 mL, i.p.)	Days 15, 17, 19 and 21
CYP + CV (6%)	CV diet containing 6% CV powder	42 straight days
CYP + CV (12%)	CV diet containing 12% CV powder	42 straight days
CYP + CV (24%)	CV diet containing 24% CV powder	42 straight days

i.p.: intraperitoneal injection; CYP: cyclophosphamide; %: *w*/*w*.

**Table 3:** Primers sequence of polymerase chain reaction (PCR).

Gene	Forward	Reverse	Products (bp)
IFN-γ	CACAA GGAGG AACGC TGACT	CAGAG CAGGA TGGAA AGGCA	698
TNF-α	AGGGG ATTAT GGCTC AGGGT	CCCGT AGGGC GATTA CAGTC	626
IL-2	TCTGC GGCAT GTTCT GGATT	GAAAG GACTA GCCCA CACCC	618
β-actin	GATCG ATGCC GGTGC TAAGA	TCCTA TGGGA GAACG GCAGA	367
IL-12	TCCTC AGGGA AGATG GAGGG	GGTCA CAAGA CTACC CAGCC	365

IFN-γ : interferon-γ; TNF-α: tumor necrosis factor-α; IL-2: interleukin-2; IL-12: interleukin-12.
