# Correction: Cheng et al. Dietary *Chlorella vulgaris* Ameliorates Altered Immunomodulatory Functions in Cyclophosphamide-Induced Immunosuppressive Mice. *Nutrients* 2017, *9*, 708

**DOI:** 10.3390/nu16172867

**Published:** 2024-08-27

**Authors:** Dai Cheng, Zhaodong Wan, Xinyu Zhang, Jian Li, He Li, Chunling Wang

**Affiliations:** 1 Beijing Advanced Innovation Center for Food Nutrition and Human Health, Beijing Technology & Business University (BTBU), Beijing 100048, China; dcheng@tust.edu.cn (D.C.); lijian@th.btbu.edu.cn (J.L.); lihe@btbu.edu.cn (H.L.); 2 Key Laboratory of Food Safety and Sanitation, Ministry of Education, College of Food Engineering and Biotechnology, Tianjin University of Science and Technology, Tianjin 300457, China; wzdtust2014@126.com (Z.W.); zhangxy_0219@163.com (X.Z.)

In the original publication [1], there was a mistake in Figure 5 as published. When processing the figures in Figure 5, we mistakenly uploaded the mRNA expression levels of IL-2 as the mRNA expression levels of TNF-α. The publishing version of Figure 5C is therefore incorrect. After verification, the corrected version of Figure 5 is shown below. The only change is in the panels of Figure 5C; the rest of the figure is identical to the published version. We state that the scientific conclusions are unaffected. This correction was approved by the Academic Editor. The original publication has also been updated.

## Figures and Tables

**Figure 5:**
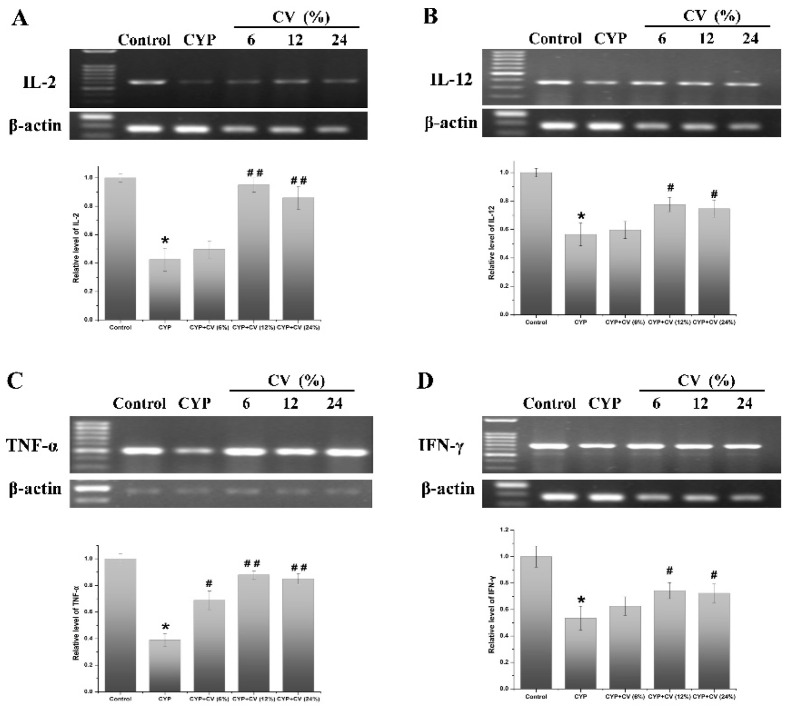
Effects of CV on the mRNA expression levels of IL-2 (**A**), IL-12 (**B**), TNF-α (**C**) and IFN-γ (**D**) in the spleen of CYP-treated mice. The β-actin was used as the control. Note: Data were expressed as mean ± SD, *: only the CYP treatment was compared against the control treatment (*p* < 0.05); #: only the CYP + CV treatment was compared against the CYP treatment (*p* < 0.05), ##: only the CYP + CV treatment was compared against the CYP treatment (*p* < 0.01).
